# Recycling of Commercially Available Biobased Thermoset Polyurethane Using Covalent Adaptable Network Mechanisms

**DOI:** 10.3390/polym16152217

**Published:** 2024-08-03

**Authors:** Edoardo Miravalle, Gabriele Viada, Matteo Bonomo, Claudia Barolo, Pierangiola Bracco, Marco Zanetti

**Affiliations:** 1 Department of Chemistry, NIS Interdepartmental Centre, University of Turin, Via Pietro Giuria 7, 10125 Turin, Italy; edoardo.miravalle@unito.it (E.M.); matteo.bonomo@unito.it (M.B.);; 2 Instm Reference Centre, University of Turin, Via G. Quarello 15A, 10135 Turin, Italy; 3 SUSPLAS@UniTo, Sustainable Plastic Scientific Hub, University of Turin, Via Pietro Giuria 7, 10125 Turin, Italy

**Keywords:** recycling, CAN, polyurethane, thermoset, Sovermol780^®^, Tolonate X FLO 100^®^

## Abstract

Until recently, recycling thermoset polyurethanes (PUs) was limited to degrading methods. The development of covalent adaptable networks (CANs), to which PUs can be assigned, has opened novel possibilities for actual recycling. Most efforts in this area have been directed toward inventing new materials that can benefit from CAN theory; presently, little or nothing has been applied to industrially producible materials. In this study, both an industrially available polyol (Sovermol780^®^) and isocyanate (Tolonate X FLO 100^®^) with percentages of bioderived components were employed, resulting in a potentially scalable and industrially producible material. The resultant network could be reworked up to three times, maintaining the crosslinked structure without significantly changing the thermal properties. Improvements in mechanical parameters were observed when comparing the pristine material to the material exposed to three rework processes, with gains of roughly 50% in elongation at break and 20% in tensile strength despite a 25% decrease in Young’s modulus and crosslink density. Thus, it was demonstrated that theory may be profitably applied even to materials that are not designed including additional bonds but instead rely just on the dynamic urethane bond that is naturally present in the network.

## 1. Introduction

Polyurethanes (PUs) are particularly versatile because of the large range of precursors, isocyanates and polyols, which can be tailored as desired. Due to these qualities, they are used in a wide range of industries, from construction materials [1] to packaging [2] and even biomedical applications [3,4], making them the sixth most manufactured polymer in the world and the first thermoset, accounting for 5.5% of all plastics produced worldwide [5]. Even with the rise of polyurethane materials, the conventional synthetic pathway of PUs (i.e., a polyaddition between polyisocyanates and polyols in the presence of a catalyst) presents some dramatic sustainability concerns. As a matter of fact, the precursors used in the synthesis of both polyols and isocyanates come mostly from petroleum-based sources, thus leading to adverse environmental impacts and poor sustainability [6]. Moreover, when dealing with thermosetting PUs, an additional issue concerns their end-of-life: the most common industrially available alternative to landfilling the large amount of thermoset PU waste (which is estimated to be between 2.1 and 3.6 million tons in Europe alone [7]) is to burn the polymer, leading to additional CO_2_ production and further negatively impacting overall sustainability [8].

In this context, the recycling of thermoset PUs is far to be obvious. Nevertheless, several reprocessing methodologies are currently being studied or used. Chemical recycling by glycolysis [9,10], acidolysis [11], or application of the transcarbamoylation reaction [12] can be used to break down crosslinks and obtain constituent prepolymers for use in new syntheses [13]. However, thermal treatments cover an important part of the end-of-life of these materials, primarily energy recovery through combustion [7], but also via pyrolysis to obtain syngas [14,15]; these processes, however, have inherent criticalities in the production of potential pollutants [16]. Despite this, about 50% of PUs at the end of their life are destined for the landfill [17], making it necessary to study new recycling methodologies that allow the material’s properties to be maintained, with the aim of real recycling in a sustainable economy.

The study of Covalent Adaptable Networks (CANs) provides new perspectives on thermoset material recycling. CANs are distinguished by the ability to be reprocessed at temperatures higher than operating temperatures, or through the application of other stimuli, in a manner similar to that of thermoplastic materials, due to dynamic exchange of bonds within the network [18,19,20,21]. Many types of functional groups, including acetals, esters, thioesters, imines, carbonates, carbamates, and urethanes, have been observed to provide this kind of dynamism [22]. With respect to polyurethanes, many studies have been carried out on their entire broad class, including polyhydroxyurethanes, polythiourethanes, polyureaurethanes, and composites, by observing their reprocessing capabilities [23]. Particularly from a green and circular economy perspective, the production of biobased lattices and their subsequent reprocessing has attracted much interest. Consequently, many new materials based primarily on bioderived polyols have been evaluated, both containing classical urethane bonds [24,25,26,27,28,29,30,31] and other types of dynamic bonds, such as hindered urea bonds [32], imine [33], carboxylates [34], and Dies–Alder adducts [35] in the presence or absence of a catalyst [36]. Usually, these materials contain mainly polyols as bioderived components, which are derived from waste or recovered materials that can be used mainly in laboratory-scale or small-scale production. In this work, in order to innovate the whole PU life-cycle, from raw materials selection to end-of-life disposal, we selected industrially available green precursors to produce a partially biobased thermosetting polyurethane network, also allowing the reprocessing of a potentially scalable industrial PU.

Recyclability was studied in hot pressing, repeating the process up to three times. The virgin material and the reprocessed ones were finally characterized via IR, DSC, TGA, DMA, swelling tests, tensile tests, and UV-Vis to observe the change in properties. Given the similarity of the formulation used here, it was possible to confirm the potential for rework of industrial-scale biobased materials.

## 2. Materials and Methods

### 2.1. Materials

Sovermol780^®^, a branched polyether/polyester polyol with a 65% biobased content with a medium hydroxyl value of 510 and a functionality equal to 3 was supplied by BASF (Milan, Italy). Tolonate X FLO 100^®^, a partially biobased aliphatic isocyanate polymer with a functionality equal to 2 was supplied by Vencorex (Grenoble, France). The UV absorber (UVA) and light stabilizer was supplied by Demak Polymers (Turin, Italy). Dimethylformamide (DMF) (≥99%, CAS 68-12-2) and dibutyltin dilaurate (DBTDL) were supplied by Sigma-Aldrich (Villanova D’Asti, Italy).

### 2.2. PU Formulation

Sovermol780^®^ was mixed with the DBTDL catalysts (0.02% *w*/*w* on PU) and with the UV absorber (1% *w*/*w* on polyol). The polyol mixture was stirred and degassed under vacuum and subsequently mixed with the isocyanate in a stoichiometric ratio (hydroxyl:isocyanate groups = 1:1). The polyurethane precursor mixture was degassed under vacuum and deposited on a polypropylene substrate and left to polymerize overnight at 50 °C in an oven. The final PU films of thickness 0.3 mm for the mechanical tests and 1.5 mm for the swelling test were obtained.

### 2.3. Reprcessing

**Figure 1:**
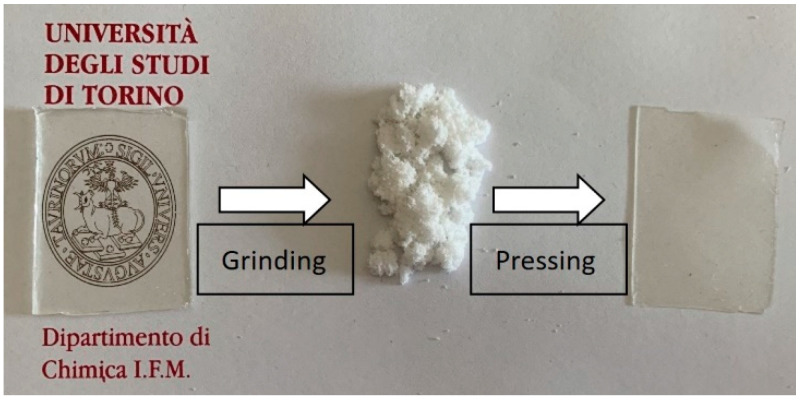
PU network through the reprocessing steps.

The reprocessing of the PU network was carried out by finely grinding the samples with a Retsch ZM 300 (Retsch, Éragny, France) centrifugal mill equipped with a 0.25 mm sieve. Samples were previously cooled in liquid nitrogen to prevent alterations due to overheating of the system. Subsequently, the obtained powder was pressed in PTFE molds for 45 min under 60 MPa of pressure at 170 °C using a hydraulic press equipped with heated plates. The films obtained had a final thickness of 0.3 mm. The scheme of the process is reported in Figure 1.

### 2.4. Characterizaion

Fourier transform infrared (FTIR) spectroscopy was used to evaluate the chemical structures of the pristine PU and the reprocessed specimens. The measurements was carried out with a Spectrum 100 (Perkin Elmer, Waltham, MA, USA) via the attenuated total reflection (ATR) technique. The spectra were measured at room temperature in the wavelength range of 4000–650 cm^−1^ with a resolution of 4 cm^−1^.

The thermogravimetric analysis (TGA) of all specimens was carried out with a TGA Q500 (TA instruments, New Castle, DE, USA) in the temperature range of 40–600 °C with a ramp of 10 °C/min under an N_2_ atmosphere with a flow of 60 mL/min on the sample. The mass of all the samples was around 12 mg.

Differential scanning calorimetry (DSC) was used to evaluate the glass transition temperature (*T_g_*) of the specimens. The analysis was carried out with a DSC Q200 (TA instruments, New Castle, DE, USA). The temperature varied between −90 °C and 60 °C with a ramp of 5 °C/min under 50 mL/min N_2_ flow, and the analysis consisted of two heating and one cooling cycle, separated with an isothermal of 5 min at the end of each cycle.

Dynamic mechanical analyses (DMAs) were carried out with a DMA Q800 (TA instruments, New Castle, DE, USA) in tension mode. The samples were cut into rectangular shapes of 20 × 5 × 0.3 mm^3^. The tests were run between 35 °C and 190 °C using a 3 °C/min ramp and 1% strain at 1 Hz with a 0.01 N preload (i.e., applied before the start of the measurement) force applied. The analyses were carried out in triplicate. The results allowed us to determine the storage modulus, and the crosslink density (*ν_e_*) was determined according to the theory of rubber elasticity for small deformations on the rubbery plateau [37,38] by applying Equation (1), *E*′ being the storage modulus in MPa, *R* the molar gas constant (8.314 J mol^−1^K^−1^), and *T_α_* the glass transition temperature determined via DSC in Kelvin. The determination of *ν_e_* was made at *T_α_*_+70_, as reported in Equation (1), as the temperature was selected in a range in which the module was not subject to fluctuations and thus in the rubbery plateau.

(1) $$\upsilon_{e}=\frac{{E\prime }_{T_{\alpha +70}}}{3RT_{\alpha +70}},$$

Stress relaxation tests were conducted with the same instrument and samples of the same dimensions at different temperatures (i.e., 160 °C, 170 °C, and 180 °C). Once the desired temperature was reached, an isotherm of 5 min was applied before applying a constant deformation of 5% for 2700 s. The stress relaxation of a dynamic crosslinked network can be defined using the Maxwell model reported in Equation (2). According to this model and to the definition of relaxation time (τ*), the time required for the sample to reach the value of 1/*e* of the initial value of the elastic modulus [21], it was possible to determine the activation energy (*E_a_*) of the networks using the Arrhenius plot, Equation (3).

(2) G(t)G0=e−tτ*,

(3) τ*=τ0eEaRT,

where *G* is the relaxation modulus in MPa, *τ* is the time in seconds, *τ*_0_ is the preexponential constant, *E_a_* is the activation energy in KJ/mol, *R* is the molar gas constant, and *T* is the absolute temperature.

Uniaxial tensile measurements were conducted on a Instron 3365 (Instron, Glenview, IL, USA) equipped with a 10 N load cell at room temperature. The specimens were cut into dog-bone shapes with dimensions according to ASTM D638 type V. The tests were conducted at 5 mm/min displacement after applying a preload force of 0.06 N, with a displacement of 2.5 mm/min. For each sample, five specimens were tested.

The UV-Vis spectra were performed with an Agilent Cary 5000 spectrophotometer (Agilent, Santa Clara, CA, USA) with wavelengths ranging from 2500 nm to 200 nm and a scanning speed of 5 nm per second. Measurements were made in the solid-state mode.

The swelling measurements were performed in DMF [39]. PU films (3 cm × 1.5 cm × 1.5 mm) were placed in a closed vial with DMF (30 mL) at room temperature. The measurements were carried out after 24 h by removing the sample from DMF and fast drying the excess of solvent with paper towel. The percentage of swelling (S%) was calculated using Equation (4):

(4) $$S\%=\frac{W-W_{0}}{W_{0}}\cdot 100,$$

where *W*_0_ and *W* are the sample weights before and after swelling, respectively. The samples were then dried in a vacuum oven for 24 h at 120 °C and weighed to assess the loss in weight:

$$\%Weight\;loss=100-(\frac{W_{D}}{W_{0}}\cdot 100)$$

where *W_d_* is the sample weight after drying.

## 3. Results and Discussion

### 3.1. Characterization of Biobased PU Network

**Figure 2:**
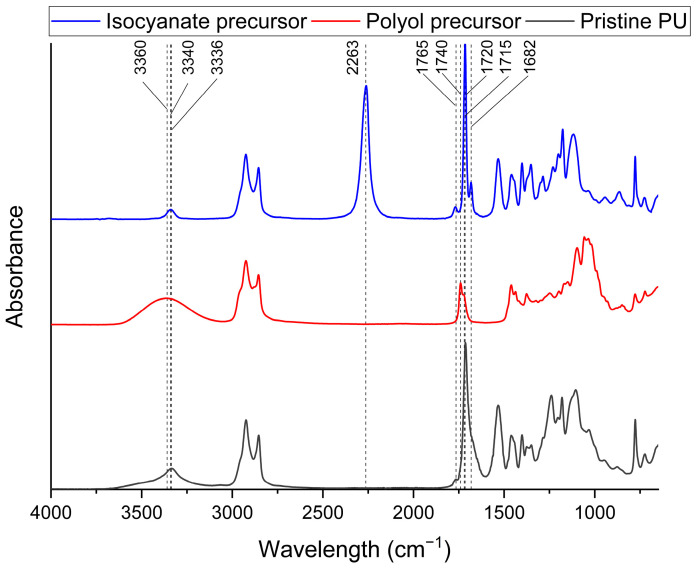
FTIR-ATR spectra of polyol and isocyanate precursor used and the pristine PU network.

**Figure 3:**
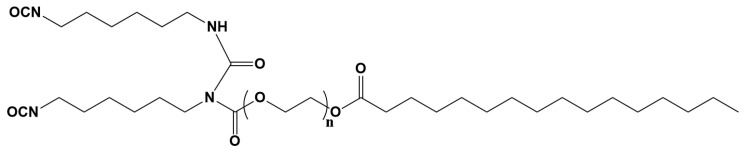
Structure proposed for isocyanate precursor, Tolonate X FLO 100 [42].

The polymerization reaction was monitored by means of FTIR-ATR spectroscopy. Figure 2 shows the spectra of the precursors and the polymer obtained. The polyol shows the characteristic broad band of -OH groups at 3360 cm^−1^ while the diisocyanate shows a sharp band around 2263 cm^−1^ [40,41]. The isocyanate shows a peak at 3340 cm^−1^, associated with N-H vibration. In the carbonyl area, different peaks can be observed for both the polyol and isocyanate. The polyol shows peaks at 1740 cm^−1^ and 1720 cm^−1^, associated with the presence of ester groups. The isocyanate instead shows peak at 1770 cm^−1^, 1715 cm^−1^, and 1682 cm^−1^, consistent with the chemical structure of Tolonate X FLO 100^®^ reported in Figure 3, which has three different carbonyl moieties, as already reported by Morales-Cerrada et al. [42]. In particular, the most intense peak at 1715 cm^−1^ can be associated with the urethane-like carbonyl, the peak at 1682 cm^−1^ to the carbonyl of the urea, and the one at 1770 cm^−1^ to the ester. In the spectrum of pristine PU, no peaks at 3360 cm^−1^ and 2260 cm^−1^ are present. The vibration of the N-H groups can be observed at 3336 cm^−1^, a typical range for H-bonded N-H groups [43]. In the carbonyl region, a broad peak at 1713 cm^−1^ is present with a shoulder at 1679 cm^−1^ and a less intense peak at 1765 cm^−1^. The absence of peaks associated with the -OH and -NCO groups confirms their total reaction and the formation of the network. With the broadening of the peak, it is no longer possible to clearly distinguish carbonyl of the urethane and urea of the isocyanate precursor. It is fair to assume that this broadening is due to the appearance of the signal attributable to the urethane crosslink, as it is consistent with the values observed in the literature for other PUs synthesized with the same mixture of isocyanates [42,44]. Meanwhile, the peak at 1765 cm^−1^ can be assigned to the carbonyl of the ester group of the isocyanate.

**Figure 4:**
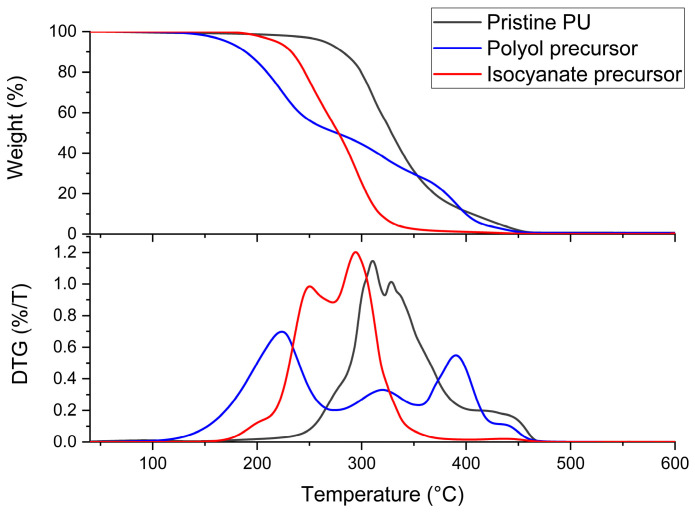
TGA and DTG profiles of precursors and the PU network.

The thermal stability of the network was analyzed by TGA. The resulting thermograms and the corresponding derivative curves for the precursors and the pristine material are reported in Figure 4. The polyol volatilizes completely between 100 °C and 460 °C, showing three stages of weight loss, as evidenced by DTG. Concerning the isocyanate precursor, volatilization starts around 190 °C and ends at 470 °C with two steps. The degradation of the PU network has its onset at 215 °C and continues up to 460 °C, when all the polymer is completely volatilized. Three different steps of degradation can be observed, with respective maximums in the derivative curves at 300 °C, 313 °C, and 387 °C. Sovermol 780 is known to be a trifunctional polyether/polyester polyol 65% bioderived; the first two degradation steps could be associated with the degradation of the bioderived component, as reported in the literature for other biobased polyols [45,46], while the third one could be associated with the remaining nonbioderived component. Regarding the isocyanate precursor, the first degradation stage can be linked to the degradation of the urethane and urea bonds and the second to the remaining part. The degradation temperature range of the PU network is comparable with other thermoset polyurethanes made with a similar composition, the same isocyanate, and different biobased polyols [42]. The pattern observed is typical of polyurethane networks since it is known to be in two or three steps. The first was linked to the degradation of the urethane bond and the subsequent formation of isocyanate and alcohol. The second, and if present the third, are instead linked to the degradation of the larger and soft segments of the polyols [47,48]. In addition, it is not possible to appreciate the formation of residues; it can be concluded that the synthesized network is not char former, and also, the amount of residual catalyst used in synthesis is not detectable by this technique.

DSC analysis was used to determine the *T_g_* of the network (thermograms reported in Supporting Information, Supplementary Figure S1). In the pristine material, the *T_g_* was observed to be −28 °C, slightly higher than that observed in similar PUs [42,44].

**Figure 5:**
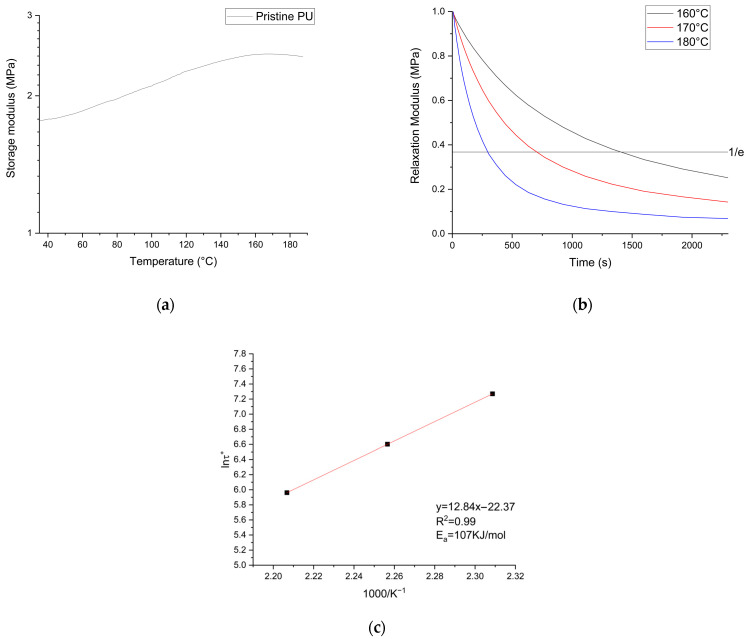
(**a**) Storage modulus of the pristine network in the range of 40–190 °C; (**b**) stress relaxation curves of the pristine network at 160 °C, 170 °C, and 180 °C; (**c**) Arrhenius analysis of the pristine network.

DMA analyses were performed to determine the storage modulus and the stress relaxation behavior of the pristine material; the results are shown in Figure 5a and Figure 5b, respectively. The modulus was shown to be constant in the range of 40 °C to 60 °C, and subsequently an increase was observed. For this reason, the modulus has been determined at *T* = *T_α_*_+70_, where *T_α_* is identified with *T_g_* calculated by DSC measurements, leading to a value of *E*′ = 1.77 ± 0.02 MPa and a calculated crosslink density of *ν_e_* = 224 mol/m^3^. These values are consistent with those observed in other thermoset PUs obtained with the same mixture of isocyanates [42]. Stress relaxation tests were performed at three different temperatures. In all cases, it is possible to observe the decrease of the value of *G*(*t*)/*G*(0) below the established value of 1/*e* for the determination of τ*. Furthermore, as the temperature increases, it is possible to observe how the value of τ* decreases, from 24 min to 12.3 min to 6.5 min, respectively, for 160 °C, 170 °C, and 180 °C. Stress relaxation analyses confirmed the dynamic properties of the system under investigation. Using the values of τ* in the Arrhenius plot, reported in Figure 5c, it was possible to determine the activation energy, obtaining a value of *E_a_* = 107 KJ/mol for the pristine system; similar values have been observed for other biobased networks [27,28,29]. With the information obtained in this phase, both the temperature and the timing of the reprocessing of the system were identified. It was chosen to use three times the value of τ* for the rework of the materials [49]. Therefore, a temperature of 170 °C was chosen with a time of 45 min under 60 MPa of pressure. The conditions were chosen to ensure the relaxation and mobility of the network at a temperature lower than the degradation temperature but high enough to minimize the reprocessing time. The resulting rework conditions were not significantly different from previous polyurethane systems, both biobased and standard [29,34,35,50,51]. Tests with lower timings and different pressures were performed, but homogeneous films could not be obtained. The time chosen is critical to the occurrence of the exchange reaction, as is the pressure required for the proper cohesion of the particles of the chopped material for film reformation.

### 3.2. Netwok Reprocessing

**Figure 6:**
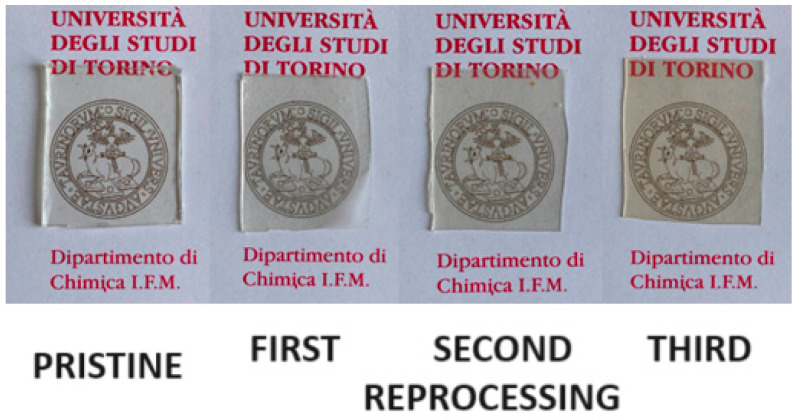
Comparison between the pristine network (first image) and the first, second, and third reprocessing (following images).

In Figure 6, the samples obtained after one, two, and three processing steps are shown, together with the pristine material for comparison. As can be observed, the specimens appear homogeneous while maintaining a very good transparency. Only after two processing steps is a slight yellowing noticeable (UV-Vis analysis reported in SI, Supplementary Figure S2).

**Figure 7:**
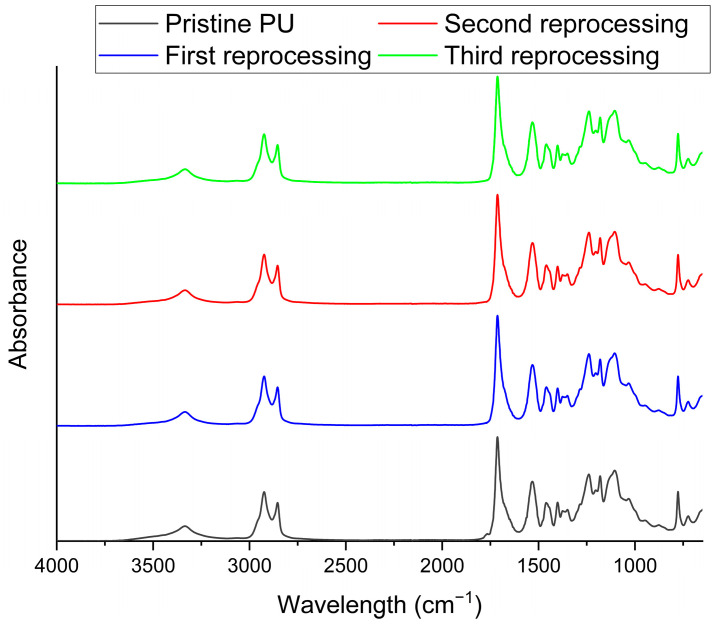
FTIR-ATR spectra of the pristine material and reprocessed samples.

**Figure 8:**
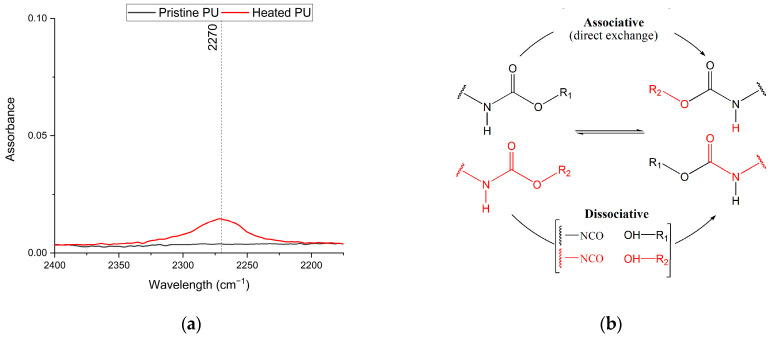
(**a**) FTIR-ATR spectra of the pristine network and the same network heated to reprocessing temperature; (**b**) urethane exchange mechanisms proposed.

The materials reprocessed after each step were characterized by means of IR spectroscopy, UV-Vis, TGA, DSC, DMA, and tensile and swelling tests and compared with the pristine network. The results of the FTIR-ATR are reported in Figure 7, in which the spectra of the three reprocessed samples and the pristine sample are reported. The spectra are identical, indicating that there are no substantial modifications in the chemical composition of the polymer. However, it is interesting to note that by recording a spectrum on the sample just extracted from the hot press, a weak band around 2273 cm^−1^ can be observed, denoting the presence of isocyanate groups (Figure 8a). As soon as the sample cools to room temperature, the isocyanate group disappears, and the observed spectrum is that shown in Figure 7. These data strongly suggest the opening of the urethane group as a result of pressure and temperature, leading to the breakdown of the network, which allows the polymer to flow as a thermoplastic. Once temperature and pressure stimuli are removed, the isocyanate group can react with the free hydroxyl groups deriving from the opening of the urethane groups to rebuild the network in the new morphology conferred on the sample, as schematically reported in Figure 8b.

The opening and closure of the network can occur in two different ways based on the exchange mechanism involved, which can be either associative or dissociative, and allows the subdivision of CANs. In networks in which the associative mechanism takes place, there is no opening of the bonds, and the exchange of chains takes place directly; while in the dissociative case, there is the opening of the bond in the constituent components followed by a phase of reformation of the new bond between different chains [52]. Observing the formation of isocyanate groups upon heating of the material leads to a confirmation that the dissociative mechanism is likely the one responsible for the rearrangement of the network, since the use of FT-IR to confirm the rearrangement mechanism has been already reported in model compounds [31] and final networks [32,53,54].

**Figure 9:**
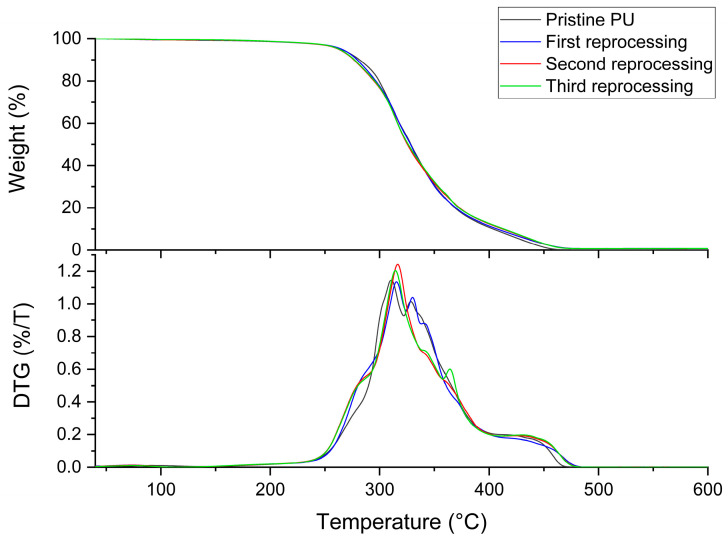
TGA and DTG profiles of the pristine network and reprocessed samples.

The TGAs of the samples are shown in Figure 9. Degradation of all samples takes place between 215 °C and 480 °C. The degradation profile of the reprocessed materials remains similar to that of the original polymer. The only substantial difference is related to the first degradation step, more pronounced in the reprocessed materials, as confirmed by the shoulder in DTG and a weight-loss temperature shifted by approximately 7 °C, as reported in Table 1. This behavior can be linked to the degradation that takes place during the reworking phase; the reprocessed material may be characterized by a higher amount of smaller fragments and a different configuration of the network, which can lead to a lower degradation temperature. In Table 1, the *T_g_* of the samples is also reported, and as can be observed, it remains almost unchanged within the experimental error (thermograms reported in Supplementary Information, Supplementary Figure S1). The value of *T_g_* depends on the degree of crosslinking; the higher the *T_g_*, the higher the crosslink density of the system [55]. Observing its stability in the reprocessed materials, it can be assumed in the first instance that the crosslinking value remained unchanged, or very similar, after reprocessing.

**Figure 10:**
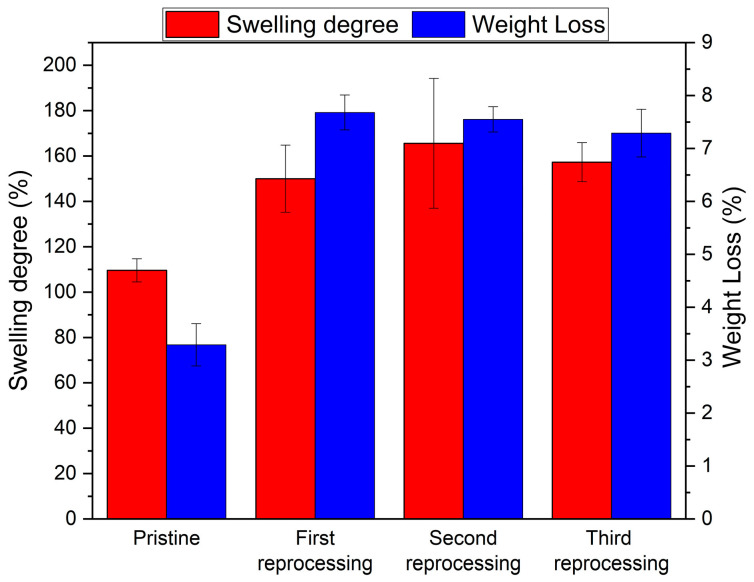
Swelling degree and weight loss of the pristine network and reprocessed samples.

To have a more quantitative evaluation of the degree of crosslinking, we performed some swelling measurements in DMF. After 24 h of immersion, one can observe substantial differences between the pristine sample (Test 1: average %S around 110%) and the three reprocessed ones (% S averages ≥ 150%) (see Figure 10). From the swelling tests, it appeared clear that the pristine polyurethane absorbs a lower amount of solvent than the reprocessed counterparts, indicative of slight loss in crosslinking density due to the reprocessing of the material. Very interestingly, the three successive reprocessing steps do not meaningfully impact the %S values, with the three samples showing comparable swelling degrees (%S averages: 150%, 165%, and 157%) within the experimental error. Following on from that, we can assume that only the first reprocessing step caused an irreversible modification of the PU network, whereas the second and the third ones seems to not have any effect on the polymer, thus paving the way for multiple reprocessing steps. It should be noted that during the swelling process, the gel (i.e., not polymerized) fraction of the polymer could be extracted by the solvent and solubilized in the bulk of the solution. The evaluation of the gel fraction (and the comparison between pristine and reprocessed samples’ values) is very important because it could give justification for the differences observed. The gel fraction could be measured by the weight difference between the pristine PU and the dried one (in an oven at 120 °C for 24 h until a constant weight was achieved) after the swollen solvent is removed, assuming that only the gel fraction is dissolved in DMF.

Upon complete evaporation of the DMF, an average weight loss of 3.4% was observed for the pristine PU (Test 1), while for the three reprocessed samples, the average weight loss ranged between 7.3% and 7.7% (see Figure 10), demonstrating a higher degree of crosslinking for the pristine PU compared to the reprocessed counterparts and proving some irreversible changes in the polymer network only in the first reprocessing step. Therefore, the pristine PU exhibits the highest crosslinking density, consequently displaying the lowest percentage of weight loss compared to the three samples obtained from the subsequent reprocessing iterations. The above-discussed data point toward the transition of 4% of the polymer network into a gel phase during the first reprocessing step, likely due to the combination of applied temperature and pressure. Indeed, a higher degree of gel phase (preserved in the other reprocessed samples) is required to ensure the reprocessability of the thermoset PUs.

**Figure 11:**
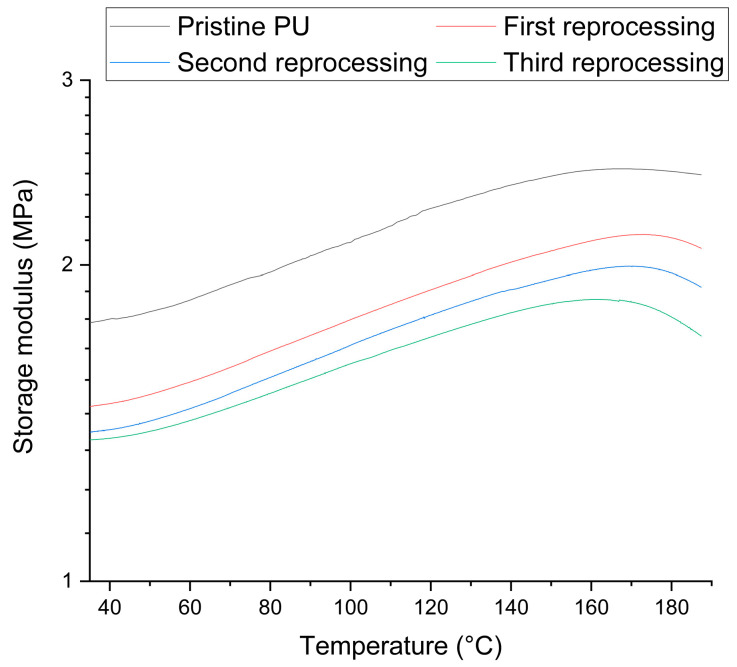
Storage modulus of the pristine network and reprocessed samples in the range of 40–190 °C.

DMA tests were conducted to obtain more accurate information on the trend of the crosslinking density of the system. The storage modulus (Figure 11) was determined for the pristine network as showing comparable values of *E*′ and ν_e_. The *E*′ values were 1.54 ± 0.10 MPa, 1.41 ± 0.02 MPa, and 1.34 ± 0.01 MPa for the first, second, and third reprocessing, respectively. The values obtained for *ν_e_* were revealed to be 197 mol/m^3^, 181 mol/m^3^, and 175 mol/m^3^ for the first, second, and third reprocessing, respectively. The trend revealed with the swelling test was confirmed; given a value of *E*′ = 1.77 ± 0.02 MPa and *ν_e_* = 224 mol/m^3^ for the pristine PU, a marked decrease in both storage modulus and crosslink density can be observed compared to that of the pristine material, but markedly, only after the first recycling process. With the subsequent steps, however, the decrease is confirmed, but in a less important and almost negligible way between the second and third reworkings. This confirms that the major changes to the system in terms of crosslinking density are made significantly only after the first step. It can be assumed that the reprocessing process causes a degradation of the network in terms of crosslink density, especially in the first step. In the subsequent steps, the diminution of the storage modulus is not as obvious, but a decrease is observable at high temperatures, a symptom of loss of mechanical properties in a more pronounced manner by increasing rework steps. In addition, through DMA it is possible to more accurately appreciate the decrease in the crosslink density of the systems due to the greater precision of the technique than swelling.

**Figure 12:**
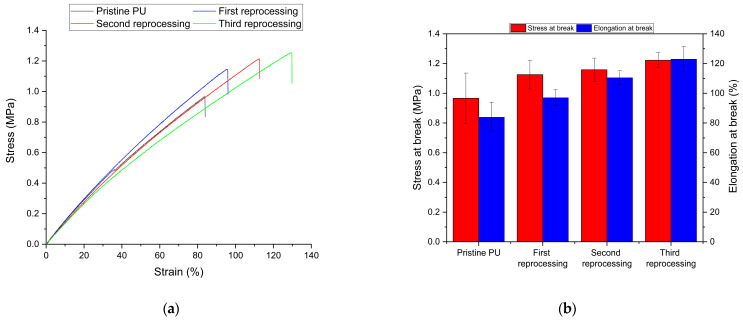
(**a**) Representative stress–strain curves for each sample, (**b**) values of stress and elongation at break for each sample.

Tensile tests were performed to evaluate the variation in mechanical properties of the reprocessed materials compared to the pristine. Representative curves for each sample and stress and elongation at break are shown in Figure 12a and Figure 12b, respectively. The results obtained for the elongation at break were 80.2% for the pristine material, 97.1%, 110.5%, and 123% for the first, second, and third reprocessing, respectively, while the tensile strengths were 0.99 MPa for the pristine, 1.09 MPa, 1.14 MPa, and 1.17 MPa for the first, second, and third reprocessing, respectively. As observed for crosslink density, there was a more pronounced increase in tensile properties, mainly from the pristine material to the first reprocessed sample. However, subsequent reworkings showed a steady increase in them, leading to statistically significant differences in the third reworked. Improvement in tensile properties has already been observed for biobased polyurethane networks. In particular, an increase in tensile strength has been observed, which has been traced to the occurrence of a significant phase separation between the soft polyol and hard isocyanate regions of the reprocessed materials [26]. Furthermore, the increase in elongation at break reported in this study can be attributed to a decrease in crosslink density, since it is known that the higher the crosslink density, the lower the elongation at break [56].

## 4. Conclusions

From a circular and sustainable economic standpoint, the demand for large-scale manufacture of new types of bioderived polymers is rising, as well as the reduction of waste and increased plastic recycling. This work demonstrated that it is possible to create a thermoset polyurethane network with a high bioderived percentage using currently accessible industrial components that can be reworked without losing mechanical qualities. The synthesized material proved to be reworkable up to three times; although a slight yellowing was observed during the rework steps; on the other hand, IR analyses did not lead to the observation of changes in its chemical composition or even the occurrence of oxidation processes in the system. Furthermore, analyses conducted by TGA and DSC showed no major changes in the thermal properties of the network. Interestingly, tensile analyses showed an increase in tensile strength and strain at break, rising, respectively, from 0.99 ± 0.12 MPa and 80.2 ± 11% for the pristine network to 1.17 ± 0.05 MPa 123.01 ± 8.4% for the third reprocessed material. The swelling test showed an increase in swelling degree, passing from 110% to 157% from the pristine to the third reworked sample, symptomatic of a decrease in crosslink density; this was also confirmed by DMA analysis, which revealed a decrease in storage modulus from 1.77 ± 0.02 MPa to 1.36 ± 0.01 MPa, respectively, for the pristine material and third reprocessed material, and consequently a lower crosslink density of 226.5 ± 3 mol/m^3^ for the pristine and 174.6 ± 1.5 mol/m^3^ for the third reprocessed material. In situ observations of the obtained network of NCO-related peaks via ATR FT-IR allowed us to gain information about the dissociative mechanism that allows the reprocessing of the material. This work allowed us to test the CAN theory as a recycling method for a novel, highly biobased PU thermoset material based on industrial available precursor. In this way, we conclude that this theory may also be applicable outside of lab-scale created materials, making this technique more appealing for prospective industrial recycling applications.

## Figures and Tables

**Table 1:** Resumptive of thermal and mechanical properties of the investigated samples.

Sample	*T_d_*_5%_ (°C)	*T_g_* (°C)	*E*′ (MPa)	*V_e_* (mol/m^3^)	Tensile Strength (MPa)	Strain at Break (%)
Pristine	266.4	−28.0	1.77 ± 0.02	226.5 ± 3	0.99 ± 0.12	80.2 ± 11
First Reprocessing	259.4	−27.6	1.54 ± 0.1	197.2 ± 12.2	1.09 ± 0.13	97.1 ± 5.5
Second Reprocessing	259.4	−28.2	1.41 ± 0.02	181.1 ± 3.1	1.14 ± 0.08	110.5 ± 4.6
Third Reprocessing	258.8	−28.3	1.36 ± 0.01	174.6 ± 1.5	1.17 ± 0.05	123 ± 8.3

## Data Availability

The original contributions presented in the study are included in the article/Supplementary Material, further inquiries can be directed to the corresponding author/s.

## Supplementary Materials

The following supporting information can be downloaded at: https://www.mdpi.com/article/10.3390/polym16152217/s1, Figure S1: DSC of pristine network and reprocessed samples; Figure S2: UV-Vis spectra of pristine network and reprocessed samples.
