# Supplementary material for: Recycling of Commercially Available Biobased Thermoset Polyurethane Using Covalent Adaptable Network Mechanisms
Source: Polymers (Basel). 2024 Aug 3;16(15):2217. doi: 10.3390/polym16152217 (PMC11314662; doi:10.3390/polym16152217)
Supplement: Supplementary file 1 [file polymers-16-02217-s001.zip › polymers-3040085-supplementary.pdf]

Recycling of Commercially Available Biobased Thermoset Polyurethane Using Covalent Adaptable Network Mechanisms

Edoardo Miravalle [1], Gabriele Viada [1], Matteo Bonomo [1,2], Claudia Barolo [1,2], Pierangiola Bracco [1] and Marco Zanetti [1,2], *

[1] Department of Chemistry, NIS Interdepartmental Centre, University of Turin, via Pietro Giuria 7, 10125, Torino (Italy)
[2] Instm Reference Centre, University of Turin, Via G. Quarello 15A, Turin, 10135, Italy
* Correspondence: marco.zanetti@unito.it

Supporting information

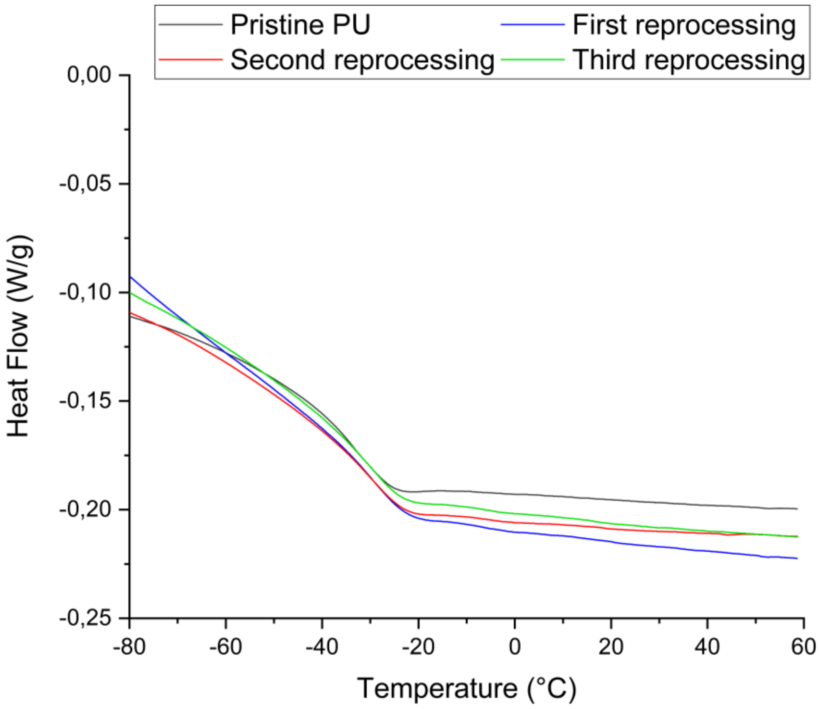

Supplementary Figure S1 DSC of pristine network and reprocessed samples.

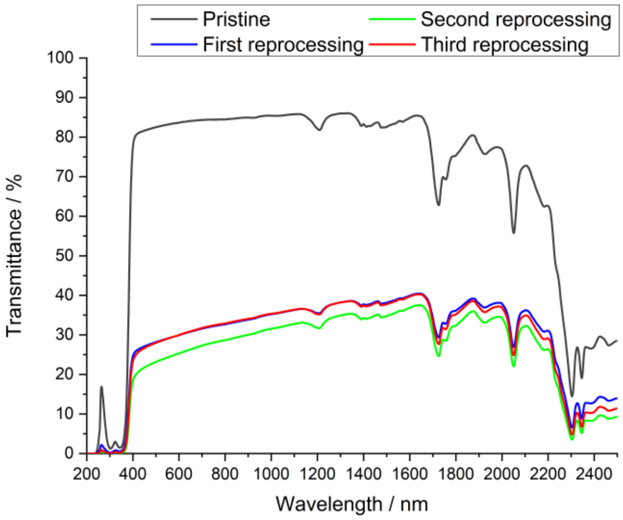

Supplementary Figure S2 UV-Vis spectra of pristine network and reprocessed samples.

Given the transparent nature of the initial biobased network and the visible yellowing of the system during rework, the behaviour of UV-vis was also investigated. Polyurethane films with a thickness of 0.3 mm were subjected to UV-vis spectroscopy analysis. Figure S2 illustrates the progressive reduction in transparency as a result of successive reprocessing steps applied to the polyurethane material. Notably, a substantial transmittance loss is observed after the initial reprocessing, while subsequent reworking does not yield significant decreases in transmittance. Across all polyurethanes, a drop is observed around 380 nm. However, the amount of UV absorbers proves insufficient for the pristine polyurethane to fully absorb radiation within the UV spectrum as depicted in Figure S2 (peaks at 270 nm and 330 nm), attributed to the thinness of the sample and its pronounced transparency. However, this behaviour is not observed for reprocessed polyurethanes, where film opacity prevents such trends.
